# Right-sided Aortic Arch with Aberrant Left Subclavian Artery in a Pregnant Female: A Case Report and Literature Review

**Published:** 2020

**Authors:** Angelina Zhyvotovska, Denis Yusupov, Rishard Abdul, Harshith Chandrakumar, Angeleque Hartt, Khaleda Akter, Yusra Qaiser, Samy I Mc Farlane

**Affiliations:** Department of Medicine, SUNY- Downstate, Health Science University. 450 Clarkson Avenue, Brooklyn, NY 11203

**Keywords:** right-sided aortic arch with aberrant left subclavian artery, right aortic arch, congenital abnormalities, aberrant left subclavian artery, vascular ring

## Abstract

Right-sided aortic arch with aberrant left subclavian artery is a rare variant of vascular anatomy. Three types of right-sided aortic arches are described and classified based on the arrangement of the aortic arch vessels, the presence or absence of congenital heart abnormalities, the relationship of the aortic arch to the trachea and esophagus, as well as the presence or absence of a complete or incomplete vascular ring. On review of the existing literature, 31 case reports were found with a spectrum of clinical presentation sand outcomes. In this case report, we highlight a case of a young female in her early 20’swho presented with choking spells, shortness of breath along with intermittent dysphagia since childhood. She was otherwise healthy and pregnant at 26 weeks gestational age. A Computed tomography scan with angiography (CTA) of the thorax was performed to rule out a pulmonary embolism (PE) however surprisingly, a right-sided aortic arch with aberrant left subclavian artery was revealed instead. Subsequently, an extensive literature review was carried out to better understand clinical presentation sand treatment strategies for this rather rare disorder.

## 1. Introduction

Vascular rings can present with non-specific respiratory and or esophageal symptoms, most commonly identified in children. Several reports has also documented symptomatic vascular rings in adults. This case report will discuss aortic arch anomalies and will emphasize the necessity of maintaining a broad differential when facing shortness of breath. Right-sided aortic arch is a rare anatomical variant present in about 0.1% of the adult population [1,2,3]. It is usually asymptomatic and diagnosed incidentally in adulthood. A right-sided aortic arch is an anatomic variant resulting from persistence of the right fourth embryologic aortic arch and involution of the left aortic arch. Normal anatomy occurs when the left aortic arch persists and the right involutes. Normally, the left subclavian artery (LSA) arises directly from the aortic arch; however, an aberrant left subclavian artery arises from the diverticulum of Kommerell, a vascular structure derived from a remnant of the fourth aortic arch. When the diverticulum of Kommerell compresses the adjacent structures, apatient may become symptomatic. To date, approximately 31 cases from the year of 2011 of right-sided aortic arch and aberrant LSA have been published in the literature [4]. The most common symptoms in this group were dysphagia, dyspnea, and cough [4]. No case reports involving pregnant females were identified by our literature review.

## 2. Materials and Methods

An electronic search was performed using the PUBMED, Medline, and Cochrane Library databases at the library of the University Hospital of Brooklyn. Five sets of search terms were used to ensure an adequate and comprehensive literature review. These included search queries ‘right-sided aortic arch’, ‘right-sided aorta’, ‘aberrant left subclavian artery’, ‘vascular ring.’

## 3. Case Report

This is a case of a 23-year-oldpregnant female at 26 weeks gestational age who presented with shortness of breath. The patient reported brief, self-resolving episodes of shortness of breath and chest tightness every other day for the past 2-3 weeks. The symptoms were worse when laying supine. She remembered having similar episodes when she was a child between the ages of 9 to 12 and several more episodes throughout her adult life. She also reported symptoms of heartburn and mild intermittent dysphagia to solidsthat have persisted since childhood. The symptoms progressively worsened with the progression of the patient’s pregnancy. The patient reported a family history of hypertension and diabetes in her mother and heart disease in her father. She denied any history of smoking, alcohol intake or illicit substance use. She reported that she lives with her parents and doesn’t communicate with the father of her child. She had poor follow up with her obstetrician and missed multiple appointments. Her only medication were Ranitidine 150 mg once daily for occasional dysphagia and prenatal vitamins.

Physical examination revealed a blood pressure of 120/75 mm Hg with a pulse rate of 75. Neck exam revealed no signs of jugular venous distension (JVD). Pulmonary and cardiovascular exam were unremarkable. Laboratory work-up was unremarkable as well.

**Figure 1.**
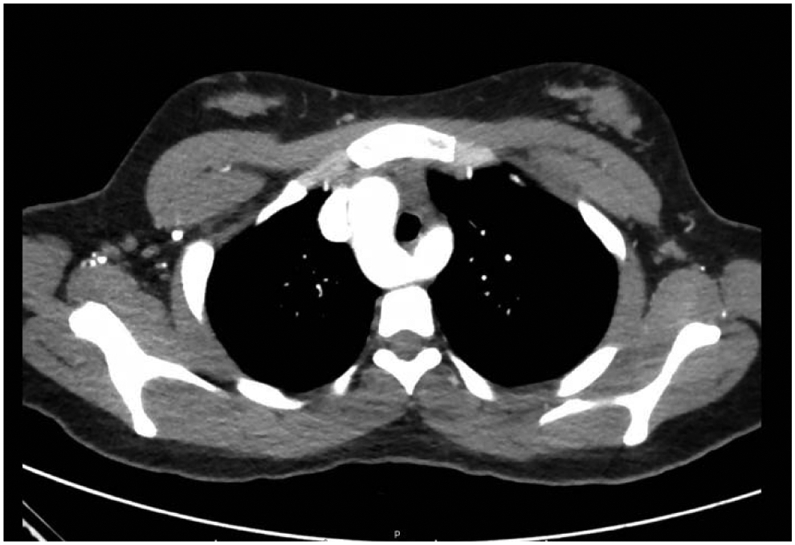
Right-sided aortic arch (RAA) with aberrant left subclavian artery with Kommerell's diverticulum (KD).

A Computed tomography with angiography (CTA) exam ruled out pulmonary embolism (PE). On close observation, a right aortic arch with aberrant left subclavian artery was incidentally discovered [Figure 1]. There was mild right-sided tracheal compression by Kommerell's diverticulum.

Over the next few days the patient’s symptoms improved, and she was discharged with close follow up with vascular surgery, gastroenterology and pulmonary clinics. Given these findings, the patient was instructed to avoid exertion, follow up with gastroenterology for a barium contrast esophagogram to detect any esophageal compression, and follow up with vascular surgery for a possible surgical intervention after the delivery. Unfortunately following this admission, the patient was lost to follow-up.

## 4. Discussion

A review of the literature published between 2011 to 2019, revealed that thirty-one adult cases of right-sided aortic arch with aberrant left subclavian artery have been reported [Table 1]. Of these, 11patients presented with dysphagia, 6 patients were asymptomatic, 2 patients presented with an aortic dissection, 2 with aneurysms, 1 with respiratory symptoms, 1 with cerebrovascular insufficiency, 1 with left upper extremity pain and numbness on exercise, 1 with left cerebral infarction, 1 with a right nonrecurrent laryngeal nerve, 1 with acute superior vena cava syndrome, 1 with pseudo-occlusion of the left internal carotid artery, 1 with subclavian steal syndrome, and 2 with rupture. The patients’ ages ranged from 23 to 80 years.

Right-sided aortic arch was first documented by Fioratti and Aglietti in 1763 [35]. In the adult population a right-sided aortic arch is often asymptomatic unless an eurysmal disease develops. The mortality associated with rupture, the morbidity caused by compression of mediastinal structures, and the complexity of surgery makes this condition clinically relevant [36]. A right aortic arch crosses the right mainstem bronchus and descends along the right side of the spine. The right-sided aortic arch is classified into three types [37,38,39]. Type I is categorized by aright-sided aortic arch with mirror image branching meaning the major arteries branching out from the arch are the left in nominate artery, followed by the right common carotid and right subclavian arteries. This type is usually associated with cyanotic congenital heart disease like Tetralogy of Fallot and truncus arteriosus [2,37,38]. Type IIis defined by a right-sided aortic arch with aberrant left subclavian artery as discussed in this case. It is associated with Kommerell’s diverticulum [39] and is rarely associated with other congenital heart diseases. Type IIIis classified by a right sided aortic arch with isolation of the left subclavian artery. In type III the left subclavian artery is connected to the pulmonary artery through the ductus arteriosus and it may be associated with subclavian steal syndrome and vertebrobasilar inefficiency. Type IIaccounts for approximately 40 % of all right-sided arches [39]. The most common is Type I [37,39].

Symptomatic formstypically present in childhood and are repaired then. In adults the symptoms may arise due to early atherosclerotic changes [40]. Dilatation of Kommerell’s diverticulum, resulting in compression of the surrounding structures, may cause shortness of breath, choking spells and dysphagia, as seen in our patient [41,42]. Other fearsome complications include aortic dissection that may present with chest pain [40]. The aberrant subclavian artery may be located posterior to the esophagus (most commonly), between the esophagus and trachea, or anterior to the trachea and it may cause compressive symptoms [1,3].

Pre-surgical evaluation of aortic arch anatomy is important for planning thoracic surgery and endovascular interventions. The presence of arch variants may influence the surgical approach taken. These arch anomalies can create complications in trans-radial coronary procedures, thoracic surgeries, and thyroid surgeries due to an abnormal recurrent laryngeal nerve course [44,45].

In pregnancy, the hemodynamic changes include increased cardiac output and expanded blood volume. Supine positioning reduces stroke volume and cardiac output and increases the heart rate due to compression of the aorta and vena cava from the enlarging uterus [46,47]. This enlargement of the uterus and compression of the vascular structures may have led to worsening of the symptoms in our patient.

## 5. Conclusion

We presented a rare case of Right-sided Aortic Arch with aberrant left subclavian artery in a pregnant female with review of the literature indicating only 31 cases reported between the years of 2011-2019in adults presenting with symptoms of a vascular ring. None of those cases involved a pregnant female. This case report and literature review highlights the importance of awareness of this diagnostic possibility among physicians, in order to broaden their differential diagnoses to include congenital anomalies, especially how subtle the findings on imaging may be. Diagnosed patients may benefit from surveillance or interventional therapy, from the prevention of misguided acute or long-term management, and avoidance of intraoperative surgical complications.

## Figures and Tables

**Figure 2.**
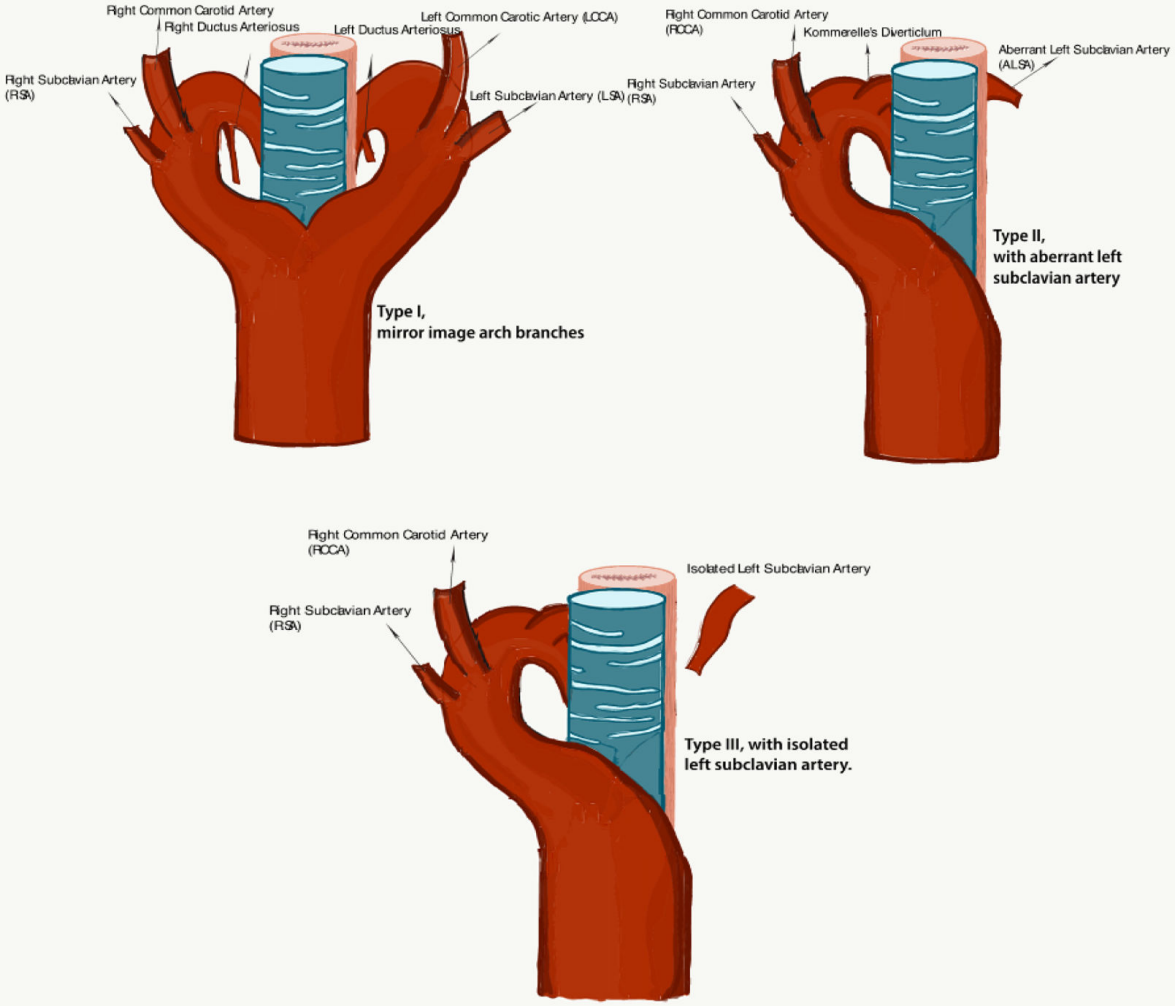
Type I: right-sided aortic arch with mirror image aortic branches; Type II: right-sided aortic arch with aberrant left subclavian artery; Type III: right-sided aortic arch with isolated left subclavian artery.

**Table 1.** Patient presentation in the studies reported in the systematic review

Case number	Year author	Reported/observed symptom/diagnostic finding
1	2019, Arakoni R [5]	Dysphagia
2	2019, Morosetti D [6]	Asymptomatic, incidental finding
3	2016, Masuoka H [7]	Right nonrecurrent laryngeal nerve
4	2019, Raymond S [8]	Dysphagia
5	2019, Morishita A [9]	Dysphagia
6	2019, Zhao C [10]	Right-sidedaortic arch aneurysm
7	2018, Tanaka Y [11]	Aneurysm arising fromKomerell’s diverticulum
8	2018, Tempaku [12]	Left cerebral infarction
9	2017, Hamady M [13]	Mild dysphagia, right-sided aneurysmal aortic arch with aneurysmal aberrant left-sided
10	2017, Wilinski J [14]	Asymptomatic
11	2016, Bhatt T [15]	Dysphagia, chest pain
12	2017, Powell BL [16]	Dysphagia with occasional regurgitation
13	2016, Ahmed MM [17]	Subclavian steal syndrome
14	2016, Lococo F [18]	Asymptomatic, incidental finding
15	2015, Parikh P [19]	Dysphagia
16	2015, Ohtani T [20]	Pseudo-occlusion of the left internal carotid artery
17	2015, Stefanczyk L [21]	Symptoms of cerebrovascular insufficiency
18	2015, Sierra-Galan LM [22]	Chronic cough
19	2014, Inui T [23]	Asymptomatic, incidental finding
20	2014, Lee CH [24]	Asymptomatic, incidental finding
21	2014, Zhang M [25]	Stanford B type dissection
22	2015, Batheeb NA [26]	Left upper limb pain and numbness on exercise
23	2014, Khalid S [27]	Dysphagia to solids
24	2013, Samas J [28]	Dysphagia
25	2013, Ebner L [29]	Dissection of the ascending aorta associated with hemopericardium
26	2013, Motoki M [30]	Ruptured aberrant left subclavian artery
27	2012, Yamashiro S [31]	Ruptured Kommerell’s diverticulum
28	2013, Suarez AE [32]	Acute superior vena cava syndrome
29	2012, Margolis J [33]	Dysphagia, voice changes
30	2011, Panduranga P [34]	Dysphagia
31	2011, Mubarak MY [3]	Asymptomatic, incidental finding
